# Uniplanar aquatic exercise quantified with inertial sensors and pose estimation

**DOI:** 10.1038/s41598-025-21131-1

**Published:** 2025-10-23

**Authors:** E. P. McShane, T. Rantalainen, M. K. Gislason, I. T. Einarsson, R. Baldvinsdottir, J. Morris, B. Wilkins, B. Waller

**Affiliations:** 1 Good Boost Ltd., Bristol, UK; 2 School of Engineering, Department of Biomedical Engineering, Reykjavik, Iceland; 3 School of Social Sciences, Department of Sport Science, Reykjavik, Iceland; 4 Finnish Institute of High Performance Sport KIHU, Jyväskylä, Finland; 5 Present Address: Henleaze House Business Centre, 13 Harbury Road, Henleaze, BS9 4PN Bristol, UK

**Keywords:** Physiology, Health occupations

## Abstract

This study presents a concurrent validity analysis of two measurement techniques for quantifying uniplanar human movement during aquatic exercise. Established marker-based biomechanical motion capture systems are labour intensive, and command significant prices, limiting their accessibility in non-specialist environments. Therefore, this study assesses the applicability of alternate measurement methods; *inertial measurement unit* (IMU) sensors and markerless *computer vision* (CV) tracking in the form of *pose estimation*, applied to the quantification and analysis of aquatic exercise. This analysis establishes the validity of each method by deriving several performance-related metrics; range of motion, duration, and angular velocity, of uniplanar aquatic exercise repetitions. Using the proposed methods it was observed that each performance metric demonstrated excellent agreement (ICC $\ge$ 0.94) across methods, based on intraclass correlation coefficients in 20 healthy young adults (n = 9 women, aged 20-26). This analysis encompassed two exercise types, knee flexion-extension and hip flexion-extension. The authors highlight that this contribution represents a step toward establishing robust, accessible methods for objective exercise quantification in aquatic settings, where previously there has been an absence of convenient technical solutions.

## Introduction

Monitoring human movement for performance analysis, injury assessment, and rehabilitation is of key importance for physiotherapists, athletic coaches, and exercise prescribers, both in conventional clinical practice and in high-performance sports^1,2^. Movement monitoring allows clinicians to gain insights based on the kinematic ability of the subject. Obtaining reliable objective performance measurements is crucial for screening, monitoring, and diagnosis, in both sports and rehabilitation contexts. Unsurprisingly, there is strong demand from the biomechanics community for accurate and convenient measurement tools^3^. A series of kinematic features capable of being used to determine the physical capacity of a subject can be extracted from inertial sensors or motion capture analysis; examples of these features include joint angles, range of motion, and segment velocities^4^. In many cases, there are well-defined outcome measures for land-based exercise and rehabilitation which utilise such metrics, such as sit-to-stand tests, range of motion assessments, and gait analysis, to name a few. Therefore, research is warranted into the usability and validity of such movement measurement tools. Aquatic resistance training and rehabilitation offers many practical benefits with respect to land-based training, for example, joint unloading; especially relevant within older populations and injury rehabilitation patients. Aquatic rehabilitation is widely implemented in clinical and community practice. Meta-analyses have demonstrated its effectiveness, with consistent improvements in mobility, balance, and pain outcomes in populations with musculoskeletal conditions and in older adults^5,6^. These findings support aquatic rehabilitation as an established, evidence-based intervention. However, despite its clinical relevance and widespread use, aquatic rehabilitation lacks objective and accessible tools for quantifying exercise performance. This limitation hinders practitioners’ ability to track progress reliably or provide performance-related feedback, reinforcing the need for convenient measurement solutions. Despite such advantages, the quantification of aquatic movement remains relatively under-explored, primarily due to technical limitations in sensing methods. Several performance-indicating parameters of aquatic exercise have been reported in the prior art, such as limb angular velocity and range of motion; when combined with limb size information, such parameters enable the quantification of tissue and muscle loading during training^7–10^. With the practical benefits of aquatic exercise in mind, it is clear that a method to obtain convenient objective measurements in this context is desirable.

Marker-based motion capture is often considered the reference standard method for quantifying kinematics (linear and angular movement of body segments), however, the technique is labour intensive and remains subject to limitations such as soft tissue artefacts^11^. Previous studies have assessed the validity of motion capture systems in above- and below-water environments versus established commercial systems; when both the camera and the body parts of interest are submerged, refraction is not a significant concern, with motion capture providing similar precision both on land and underwater^12^. However, modern motion capture systems found in places such as biomechanics laboratories command a significant price, limiting their accessibility and applicability in non-specialist environments^13^. Additionally, fluid drag may exacerbate motion artefacts compared to on-land testing. Hence, alternative methods of quantifying aquatic exercise warrant exploration. The authors note that there are examples of bespoke marker-based analysis systems designed for aquatic use (e.g., Qualisys Oqus3+ underwater), however issues with accessibility remain.

Alternatives to marker-based quantification do exist. Specifically for aquatic environments, waterproof inertial measurement unit (IMU) sensors can be used underwater^14,15^, and have been shown to be reliable and valid for movement quantification in general^16^. The cost of IMUs varies depending on performance and operational scope, however affordable options are available^17^. When mounted on a human subject, IMU measurements can provide useful data on limb movement, contributing to exercise performance assessment^18–20^. A second method of aquatic exercise quantification is motion capture analysis. Due to our desire to assess convenient solutions, within this work we are primarily interested in automatic digitisation of motion capture footage, rather than analysis based on manual labelling. Automatic or semi-automatic markerless (as opposed to marker-based) motion capture analysis systems are based upon machine-learning enabled computer vision (CV). Recently, such automated analyses have been shown to agree well with manual digitisation^21^. These systems can operate using standard optical video and do not require specialist imaging setups, making them potentially well-suited for use in aquatic environments. Markerless analysis of exercise has relatively recently been shown to have sufficient precision to be feasible for exercise monitoring^4,22,23^, therefore, we assess application in aquatic exercise quantification. Pose estimation is a subcategory of CV algorithm which is of direct relevance to this work; such models allow anatomical landmarks (known as ‘key-points’) to be detected from images of human subjects automatically^24–26^, thereby removing the need for manual digitisation for body part tracking. This method has been applied both within 2D and 3D pose estimation^27,28^, studies have used a dual-camera (stereo) perspective to transform 2D images or videos to 3D coordinate space^3,29,30^. Low-cost portable camera systems have recently been established as viable for pose estimation^31^, enhancing the accessibility of applying such methods. Here we utilise pose estimation models to detect ankle, and knee key-points from each frame of video footage containing subjects performing uniplanar exercise.

The precision and concurrent criterion validity for estimating kinematics of land-based exercise of both IMU and markerless motion capture has been previously established; we encourage the reader to consult recent reviews on the subject; IMU^32^ and markerless motion capture^33,34^. In brief, we believe it is fair to summarise the literature by stating that marker-based motion capture, markerless motion capture, and IMU-based 3D kinematic assessments agree best in major movement plane kinematics (commonly sagittal on land) estimation. However, their deduction of frontal and coronal plane kinematics leave some to be desired. Moreover, neither method (markerless or IMU-based) produces the same results as marker-based analysis, nor should they be expected to, as the segment coordinate systems between methods are not identical. As such, our study focuses exclusively on uniplanar movement, where agreement is most feasible.

This study contributes a novel assessment of IMU and CV-based measurement for aquatic rehabilitation, a previously under-explored domain. Although each of these measurement approaches have been applied to varying degrees for the quantification of land-based exercise kinematics^35–38^, a relative dearth of research is to be found for aquatic exercise, where their application remains an emerging area. Therefore, the purpose of the present study was to assess the validity of applying both movement quantification methods to water-based uniplanar exercise. The validity of each method is assessed through the deduction of several performance-related metrics; range of motion, repetition duration, and angular velocity. The agreement between measurement methods is evaluated using intraclass correlation coefficients in 20 healthy young adults (n = 9 women, aged 20-26; 22 recruited; 20 analysed for knee; 18 for hip). This analysis encompasses two exercise types, knee flexion-extension and hip flexion-extension. We hypothesize that it is unlikely that these independent quantifications agree unless both methods actually provide a reasonable quantification of aquatic exercise. By comparing the results of these independent techniques, we aim to determine whether they converge on meaningful performance metrics, and attempt to establish whether each method is viable for underwater movement quantification; where a convenient accessible solution does not currently exist.

## Results

The methodology applied to generate the following results is expanded on within the methods section. For each participant simultaneous IMU and video recording was obtained; during which time repetitions of uniplanar shank motion were performed as part of knee flexion-extension and hip flexion-extension exercises. To ensure that shank motion was representative of the intended exercise, the technique was demonstrated as a single-joint movement to minimise contributions from other joints. Video recording of two participants was unsuccessful due to poor visibility conditions and data for n = 20 is presented. Two further participants were lost to the exercising limb going out of the field of view (partially out of water) in the hip flexion-extension exercise leaving n = 18 for this movement. The mean age, height, and body mass of the volunteers (n = 20, women = 9) were 23.2(1.5) years-of-age, 179.4(5.4) cm, and 81.5(8.4) kg. In general, excellent agreement was observed between measurement methods for all assessed quantification metrics in knee flexion-extension (ICC 0.96 to 1.00, Table 1 (upper panel)) and hip flexion-extension (ICC 0.94 to 0.98, Table 1 (lower panel)).

**Fig. 1:**
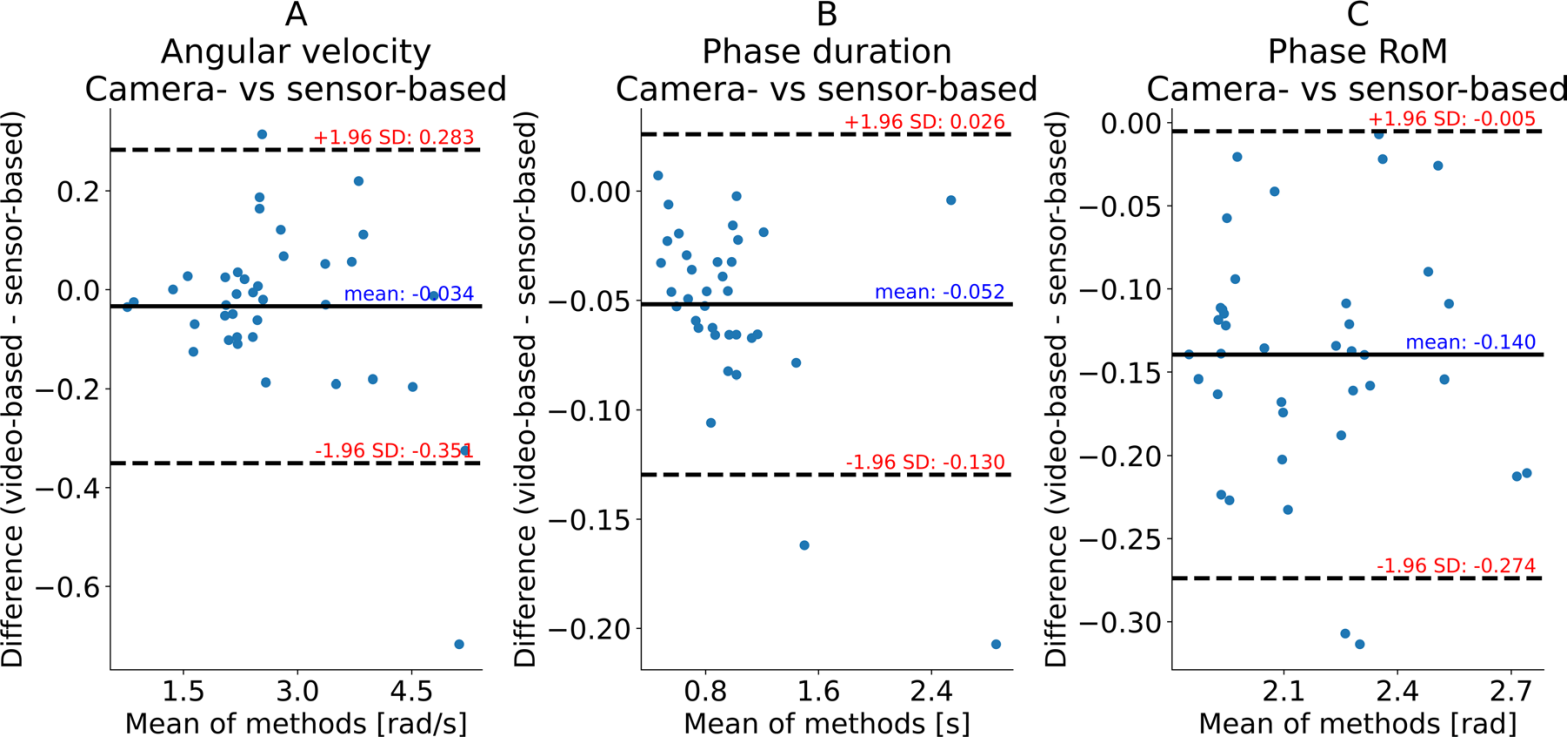
Knee Flexion-Extension: Bland-Altman plots of pose-estimation-based (camera) and inertial measurement unit (IMU)-based (sensor) shank kinematics quantifications of the aquatic knee flexion-extension exercise evaluated in the present study. Flexion and extension phases were quantified independently and pooled in the analysis. Note that the absolute values rather than signed values were used in these analyses. (**a**) Mean angular velocity of repetition phase. (**b**) Phase duration. (**c**) Range of motion of the phase.

For the knee flexion-extension exercise a mean bias between methods was observed in exercise phase repetition duration, and range of motion with the IMU-based method indicating 5.5% to 6.7% higher values (both p < 0.001) than the camera-based method (Table 1 (upper panel), Fig. 1).

**Fig. 2:**
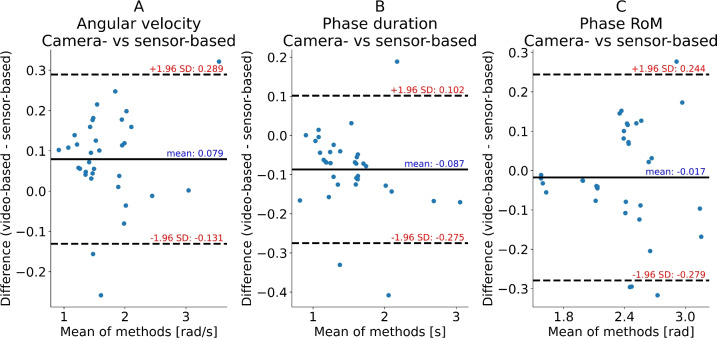
Hip Flexion-Extension: Bland-Altman plots of pose-estimation-based (camera) and inertial measurement unit (IMU)-based (sensor) shank kinematics quantifications of the aquatic hip flexion-extension exercise evaluated in the present study. Flexion and extension phases were quantified independently and pooled in the analysis. Note that the absolute values rather than signed values were used in these analyses. (**a**) Mean angular velocity of repetition phase. (**b**) Phase duration. (**c**) Range of motion of the phase.

For the hip flexion-extension exercise a mean bias between methods was observed in angular velocity and exercise phase repetition duration with the IMU-based method indicating 4.6% lower, and 5.8% higher values (both p < 0.001) than the camera-based method (Table 1 (lower panel), Fig. 2).

Normalising distributions by removing apparent outliers did not change the findings in sensitivity analyses for normality violations. Sensitivity analyses performed by removing apparent outliers did not materially affect the observed agreement, suggesting robustness of our findings despite some extreme data points.

**Table 1:** Intraclass correlation coefficients (ICC; single fixed raters for consistency; ICC(3,1)) between inertial measurement unit (IMU)-based and pose-estimation-based assessments of shank kinematics for knee and hip flexion-extension exercises. Values are given for the pooled exercise phases (flexion and extension).

Exercise	Variable	Bias (95% CI)	ICC (95% CI^a^)
Knee Flexion-Extension	Angular velocity [$^{\circ }$/s]	-4.93 to 1.07	0.99 (0.98 to 0.99)
	Repetition duration [s]	-0.06 to -0.04	1.00 (0.99 to 1.00)
	Range of motion [$^{\circ }$]	-9.27 to -6.72	0.96 (0.92 to 0.98)
Hip Flexion-Extension	Angular velocity [$^{\circ }$/s]	2.44 to 6.65	0.98 (0.96 to 0.99)
	Repetition duration [s]	-0.12 to -0.05	0.98 (0.96 to 0.99)
	Range of motion [$^{\circ }$]	-3.63 to 1.62	0.94 (0.89 to 0.97)

^a^ CI = confidence interval

## Discussion

This study explored the feasibility of using two accessible, low-cost movement quantification approaches, IMU sensors and computer vision-based pose estimation, for aquatic exercise monitoring; a domain where traditional gold-standard systems are impractical or inaccessible. In particular, we focused on the extraction of several performance-related metrics; including range of motion, angular velocity, and repetition duration. Although ICC values indicated excellent agreement ($\ge 0.94$), significant bias was observed and consequently the methods should not be considered interchangeable. Bland-Altman analyses revealed random errors (i.e., difference between concurrent assessments as indicated by the LoA) of approximately $\pm 10^{\circ }$ for ROM and $\pm 20$ deg/s for angular velocity. These values indicate that the reliability of the systems still needs be established to confirm that better than smallest clinically relevant difference can be detected. Based on the agreement and absolute congruency between the proposed measurement modalities, and the previous validation of land-based exercise monitoring using each approach, these findings support the feasibility of using either method to quantify aquatic exercise under controlled conditions. In doing so, this study contributes to addressing the long-standing gap in aquatic rehabilitation technology, by offering a framework to obtain objective performance metrics where conventional tools fall short. The accessibility of the presented quantification methods may be particularly valuable to practitioners seeking a low-cost, user-friendly tool for exercise monitoring in aquatic settings. We postulate that the benefit of applying such methods may not be in their absolute measurement accuracy, but in their ability to give convenient and rapid performance-related feedback, without the need for complex imaging setups, manual motion capture digitisation, or expensive hardware.

As mentioned in the introduction, both IMUs and machine learning-enabled CV have been shown to have excellent concurrent criterion validity to gold standard kinematics assessment methods for major movement plane kinematics (commonly sagittal on land)^35–38^. However, few such assessments have been completed underwater. We have previously applied an alternative machine learning-enabled joint tracking method (DeepLabCut) in 2D underwater analysis where we found excellent agreement between manual digitisation and the machine-labelled joint positions^21^. Kadi et al. reported a comparison of propulsive force estimation based on hand kinematics assessed with IMUs and pressure sensors on the palm against 3D motion capture and found excellent congruence between the methods in front crawl swimming based on six participants^39^. Their method can only produce comparable results between the methods if the hand orientation was accurately estimated with the IMU, and the orientation estimate depends on valid measurement of the kinematics. We were unable to identify previous research on the concurrent criterion validity between underwater markerless motion capture and IMU-based kinematics, but in our estimation the present findings of excellent congruency in uniplanar exercise are well-aligned with the body of literature regarding criterion validity of each method versus reference standard marker-based motion capture. Our findings contribute to the growing evidence base that accessible, markerless systems can be viable alternatives in environments where traditional gold-standard tools are impractical. Notably, we mitigated the challenge of poor frontal and coronal plane (abduction/adduction or external/internal rotation) kinematics by reducing the exploration to the dominant movement direction. While this approach proved successful, direct applicability to more complex multi-joint movements remains to be established.

A follow-up study could assess the validity of using the proposed measurement modalities in the context of assessments and outcome measures. The potential of these methods extends beyond simple exercise quantification. Angular velocity measurements, for instance, may allow loading estimation based on fluid dynamics principles, assuming constant limb morphology (fixed limb length). This opens the door to low-cost load monitoring during aquatic rehabilitation, an area currently underserved by available technology in a convenient and accessible way. Even if, hypothetically, absolute values differed slightly from those captured in clinical labs, the reliability (previously established on land^16^) and low setup demands make both methods appealing for tracking relative changes over time, offering potential value. For example, in a rehabilitation setting focused on post-operative knee mobility, clinicians could track the progression of limb angular velocity over time using the proposed tools, offering insights into progression. This feedback could be enhanced in scenarios where baseline measurements of pre-injury performance were available for comparison. Despite these potential applications, several limitations temper our conclusions.

Although our findings are encouraging, several limitations must be acknowledged. Firstly, one important limitation of this study is that we did not use a concurrent reference standard system due to the discussed challenges of underwater motion tracking. While marker-based motion capture is often regarded as a criterion method in biomechanics, its accuracy can be affected by soft tissue artefacts, which are particularly pronounced in aquatic environments. Instead, we assessed the agreement between two independent modalities as a proxy for validity. While this limits absolute accuracy claims, the strong congruence observed provides encouraging evidence of consistency and practical value in applied contexts. It should also be noted that our analysis quantifies shank segment kinematics, not direct joint angles. As such, compensatory strategies at the knee or hip could influence segment motion and may not directly reflect underlying joint flexion-extension. This limitation has to be overcome by controlling exercise execution and reinforces that the present study should be regarded as a proof-of-concept demonstration of feasibility rather than a ready-to-use clinical tool. As mentioned, such levels of absolute accuracy may not always be strictly necessary to provide value to physiotherapists and strength and conditioning professionals; i.e. convenient, robust, indicative measurements such as those provided here can offer relevant insights, both for establishing the current ability of a subject, and also in tracking progress over time. The technologies discussed here represent accessible and relatively cost-effective means of gaining performance insights in aquatic scenarios, hence we believe there is value in applying such approaches in the context of sports and rehabilitative practice. While the lack of a gold-standard comparison (or access to a specialised aquatic tracking system) is a limitation, it also reflects the broader technical challenges faced in aquatic biomechanics research. In such contexts, establishing validity through cross-modal agreement offers a pragmatic alternative. This study provides one such effort, showing that two fundamentally different sensing paradigms, each with their own limitations, yielded highly correlated performance metrics. Secondly, certain technical issues arose during pose estimation, including inaccurate key-point tracking in cases of poor lighting, partial body occlusion, and bubbles dragged from the surface. These resulted in data exclusion for some participants (i.e., we recruited n = 22 but report on n = 20), highlighting the sensitivity of current CV tools in aquatic environments. Although the use of MMPose yielded reasonable results in most recordings, further model optimisation, such as fine-tuning, integration of temporal smoothing, or transfer learning, could improve robustness^40^. Additionally, pose estimation failed in exercises where the limb exited the field of view or generated significant turbulence on re-entry (additional N = 2 in the hip flexion-extension exercise lost to this), suggesting hardware placement and environmental control are key considerations for future studies. Other models aside from MMPose were also explored in the initial stages of the present project (e.g., BlazePose, AlphaPose, OpenPose), however, partial body absence in the field of view resulted in failure of some of these key-point inference methods, therefore MMPose was selected as a viable option due to its more robust detection mechanism. Finally, some variables or residuals of variable pairs did not conform well with the normal distribution. As mentioned in the results, these violations of normality did not affect the overall findings. One participant performed the movements significantly slower than the rest of the cohort, which appeared as an outlier with phase durations greater than 2.5 seconds and another performed the leg swings significantly faster than the rest of the cohort. Findings remained the same in a sensitivity analysis without considering their result, confirming our primary results were not driven by outliers, maintaining high agreement between measurement systems. Another apparent outlier was evident in knee extension-flexion exercise where > 30 $^{\circ }$/s difference between methods was observed. Visual inspection of the IMU signals appeared as though the sensor may have been pinched and flicked by the fin worn to provide resistance for the exercise with conspicuous spikes visible in the recorded angular velocity not apparent in any other recording. Sensitivity analysis with this participant removed did not change the findings. We wish to reiterate that while consistency between methods was encouraging, the reliability of the methods remains to be established. That is, the LoA indicated potentially meaningfully large random error. Hence, reliability needs to be established in further research to establish whether or not reliability is better than the smallest worthwhile change relevant to aquatic rehabilitation and/or aquatic exercise monitoring.

Despite the challenges mentioned above, we claim that the present findings provide evidence towards justifying our assumption that low-cost wearable and computer vision-based technologies can provide convenient, potentially clinically relevant insights into aquatic performance. These tools could enable routine monitoring of rehabilitation progress and exercise compliance in aquatic settings, where objective metrics have traditionally been difficult to obtain. Contemporary commentaries, e.g. Taylor and colleagues (2021)^41^, highlight the utility of monitoring external and internal loads in rehabilitation. Tracking improvements and relative performance changes rather than absolute values suffices for the purpose. Thus, a consistent small systematic bias (such as observed here) would typically be acceptable as long as the bias remains stable over repeated assessments, allowing clinicians to reliably track patient progress or recovery trends.

In conclusion, we found strong agreement between the IMU and artificial intelligence-enabled CV-methods, which according to our hypothesis indicated that both methods may provide viable uniplanar exercise monitoring and quantification methods in aquatic settings. The evaluated performance metrics (angular velocity, range of motion, duration) are of interest both in patient monitoring, and in athletic performance evaluation. Pending confirmation of adequate reliability, both methods provide feasible tools for monitoring relative performance and progression in aquatic rehabilitation and training scenarios. Our findings specifically support the applicability of both IMU-based and video-based methods as objective quantification tools in aquatic exercise contexts, an area traditionally lacking robust measurement solutions. These methods provide meaningful and actionable metrics (such as segment angular velocity, range of motion, and movement consistency) relevant to therapists or coaches who previously relied primarily on subjective visual assessments. Importantly, these results suggest that either measurement approach may provide accessible options for quantifying aquatic exercise, though selection should be informed by awareness of the observed random error and the intended clinical or training context (e.g., equipment availability, ease of use, or cost). The true reliability of the methods remains to be established as well as more rigorous concurrent criterion validity evaluation. Nevertheless, the present findings provide proof-of-concept justification for further exploration of either method for performance monitoring in uniplanar single joint aquatic exercise. In summary, both of the presented IMU and computer vision pose-estimation-based quantification methods demonstrate potential for accessible aquatic exercise monitoring, particularly for tracking relative performance changes in controlled tasks. This study lays important groundwork for more robust, clinically relevant applications of IMU and computer vision-based tools in aquatic exercise contexts, where traditional motion capture remains infeasible. Future research should evaluate their clinical utility across more complex multi-joint exercises and real-world rehabilitation programs and exercise regimens.

## Methods

The present study used a cross-sectional study design aimed at evaluating the agreement between two measurement techniques by estimating concurrent criterion validity. A convenience sample of n = 22 healthy adults (n = 9 women) aged 20 to 26 years was recruited by word of mouth to volunteer as participants in the present study. Data from n = 20 individuals are presented for knee flexion-extension and n = 18 for hip flexion-extension due to data loss described below. The data presented in this study were gathered across two windows during August 2023 and March 2024 in Reykjavik, Iceland, where written consent was obtained. Inclusion criteria included being able to complete the exercises and consenting to the study. Exclusion criteria included inability to complete protocol exercises safely, e.g. due to acute musculoskeletal injuries or sickness. The study was conducted in accordance with the Declaration of Helsinki, and approved by the Reykjavik University ethical review board on 14/08/2023.

**Fig. 3:**
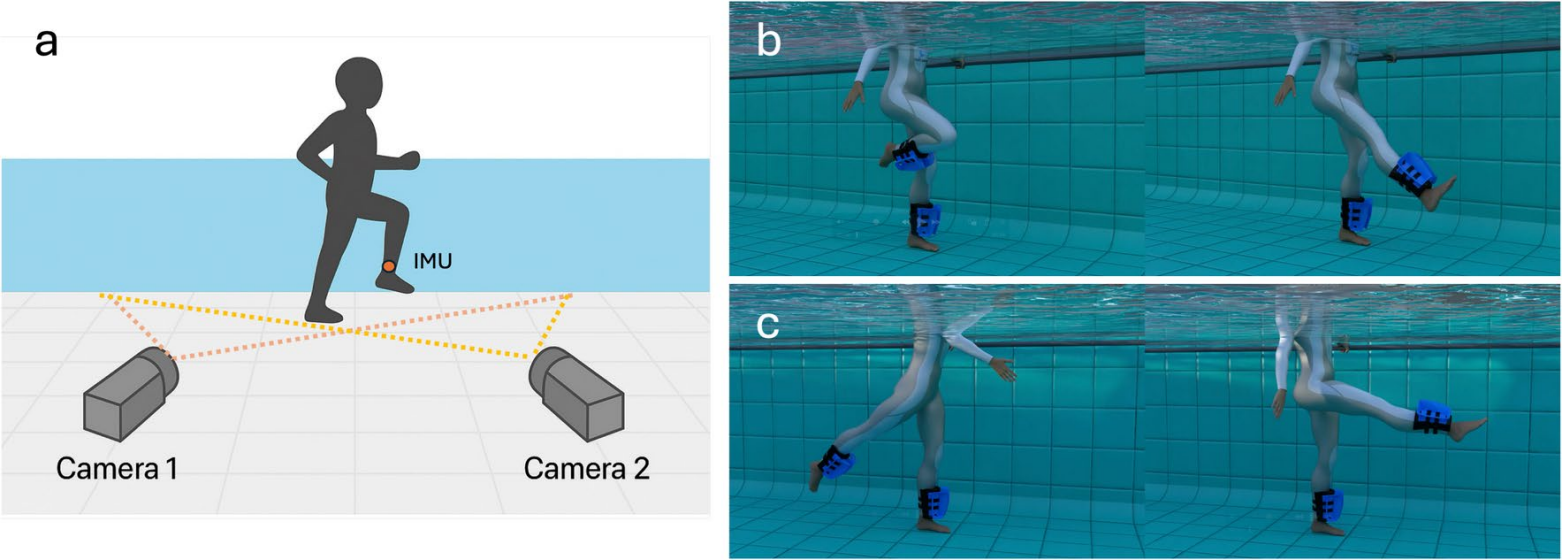
Measurement instrumentation and visualisation of the two protocol exercises. (**a**) Pool instrumentation with underwater stereo camera setup and participant-mounted IMU. (**b**) Knee flexion-extension movement with hip flexed. Participants were allowed to perform this movement using their preferred hip joint angle. (**c**) Hip flexion-extension movement with knee in full extension. These movements were demonstrated to participants prior to execution of the protocol.

**Fig. 4:**
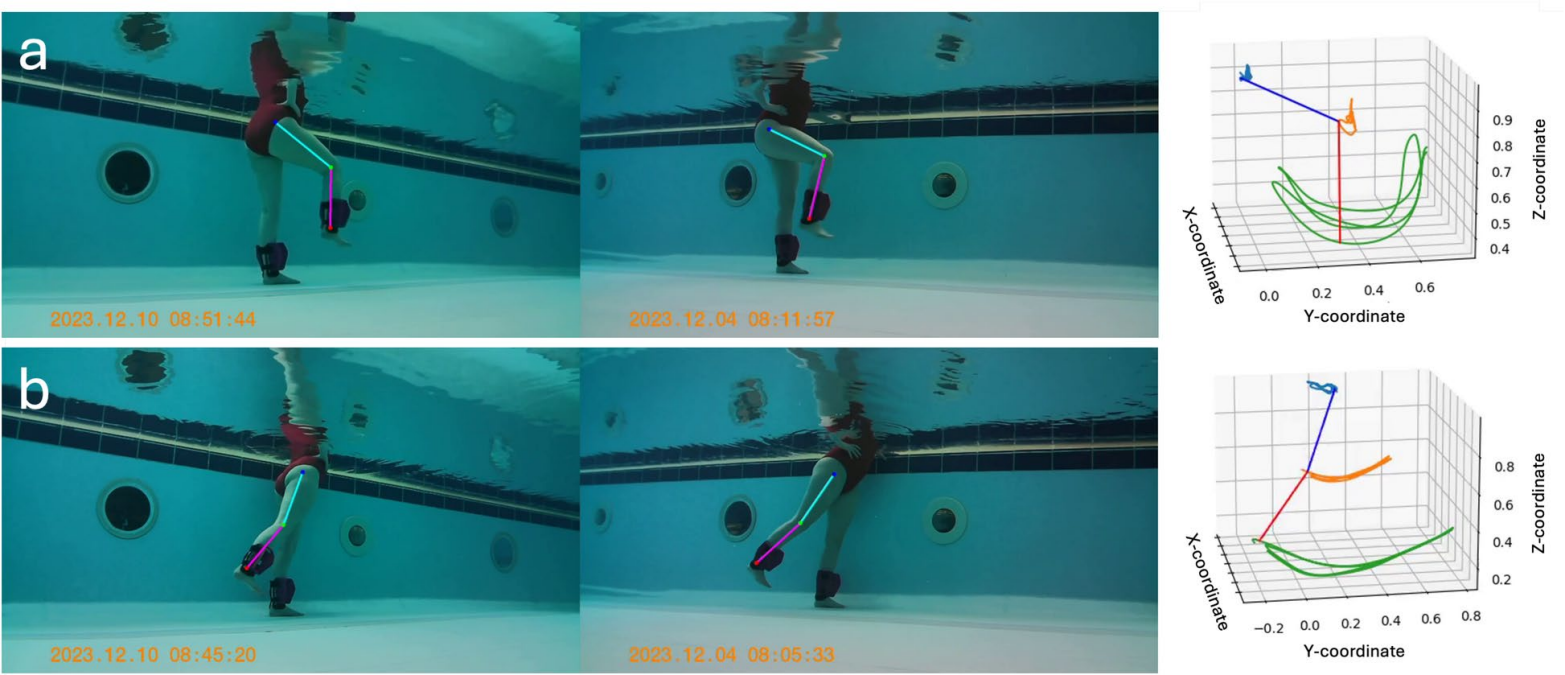
Anatomical key-points obtained via CV-enabled pose estimation and subsequent computation of shank kinematics. (**a**) Knee flexion-extension: Detected key-point 2D coordinates from two camera views are transformed into 3D coordinate space to compute shank kinematics. Ankle (green) and knee (orange) coordinates were used to compute shank kinematics; the hip (blue) key-point is shown for context. A shank vector between the ankle and knee coordinates was deduced, represented by the red line in the right-hand subfigures. (**b**) Hip flexion-extension: Identical processing conducted for second exercise type. Note: Thigh key-points are labelled here to assist with data interpretation, however, these points were not used in the subsequent analysis described elsewhere; shank kinematics are derived from knee and ankle key-points.

The pool testing area was instrumented with two underwater digital video cameras (Action Camera GA100, Wolfang, China) rigidly mounted on the floor of the pool, allowing motion capture of the exercise session from two perspectives (shown in Fig. 3a). The field of view of each camera was directed towards the testing area with the optical axes intersecting at a 60$^{\circ }$ angle. We used stereo camera configuration rather than a single orthogonal camera due to our desire to transform detected key-points into 3D space. In an on-land lab setting, one camera perpendicular to the motion plane is often sufficient for 2D analysis. However, in the aquatic environment it is challenging to ensure that the participant’s motion stays perfectly in a single plane relative to a camera, due to floating, water currents, which may cause slight changes in movement direction. Therefore, we opted for a dual-camera setup to capture the motion in 3D rather than in 2D. Video footage recorded at 60 frames per second with full HD (1920 x 1080 pixels) resolution was captured for the duration of each exercise session. Examples of the perspective of each camera can be seen within Fig. 4a,b. Prior to the participant entering the pool a three-dimensional (3D) 1 m x 1 m x 1 m calibration object with 21 control points was placed on the bottom of the pool in the exercise area to enable 3D spatial calibration during post-processing^42^.

The study protocol required participants to attend a single testing session at a 1.2 m deep indoor therapeutic pool (water temperature 30 $^{\circ }$C). Before entering the water, participants were fitted with leg-worn fins (AquaStrength, USA) to enhance workout intensity through increased drag. A single IMU sensor (Movesense Flash, Movesense Ltd., Vantaa, Finland) was mounted using Velcro straps on the right ankle just above the malleoli. The participants were then allowed to warm up *ad libitum* prior to entering the pool for testing. Upon entry into the pool, participants were directed to the specific area that had been prepared for testing, including underwater camera setup and prerequisite camera calibration. The participants were then instructed to execute bouts of each uniplanar exercise. Firstly, they were instructed to perform two sets of self-paced 30 second bouts of isolated unilateral knee flexion-extension movement with hip flexed to allow completing the exercise. No restriction on hip flexion angle was applied (or whether it changed throughout the exercise). Demonstration of the exercise was given with a relatively constant hip joint angle. Following each exercise set a period of rest was conducted, lasting one minute. Then, participants were asked to perform two sets of self-paced 30 second bouts of hip flexion-extension movement with the knee in full extension, including a rest between sets. The performance of each movement is visually represented in Fig. 3b,c.

Throughout this process, video camera footage from the two underwater cameras and IMU data from the right leg were simultaneously recorded. Upon exercise completion, participants were asked to exit the pool environment, IMU sensors were removed and data was transferred to a tablet computer. Video camera footage was collected and stored after the completion of each testing session. In this fashion, the IMU and motion capture data for all subjects was obtained for post-processing.

### Pose estimation shank kinematics

Post-processing of the obtained motion capture footage required pose estimation deduction of anatomical key-points from each video frame. The extraction of the spatial locations of these body parts resulted in a time series of the participants limb movement throughout the exercise session, allowing further training performance analysis. MMPose (version 1.3.1 executed with python version 3.8.19)^43^ was used to digitise anatomical landmarks from the recorded video footage. Pose estimation inference was conducted using ‘RTMDet’ (Real-Time Models for object Detection)^44,45^, pre-trained on the general object detection dataset ’COCO’ (Common Objects in Context)^46^; detecting 17 anatomical landmarks (key-points) located across the body. Only the knee and ankle key-points were used to compute shank kinematics; the hip key-point was displayed in Fig. 4 for anatomical context. Examples of detected key-points of interest from each camera view and each exercise type are shown within Fig. 4, where extrapolated lines between points are included. Video clips were manually trimmed to contain only the exercise set of a particular exercise (e.g. knee flexion-extension). A bespoke algorithm was developed and used on a single participant to correct mistakenly identified left and right leg digitisations.

Key-point time-series pairs from each camera were subsequently synchronised based on the x-coordinates of the ankle by a brute force search of offsets from -3 s to 3 s with an increment of 10$^{-3}$ s. The offset that resulted in the smallest sum of squared differences between the series was used as the optimisation target. The values of the second camera were linearly interpolated at the frame instants of the first camera, and the intersection of sample instants was used for determining the difference between the cameras. This procedure resulted in good synchronisation between the cameras based on visual inspection. Both knee and ankle key-point x- and y-coordinates were filtered with an 11-sample moving median filter to smooth the signals and to account for possible transient erroneous digitisations, and synchronised and resampled to 50 samples per second using linear interpolation. The synchronisation offset was applied during the linear interpolation for the second camera to implement synchronisation. Resampling to 50 samples per second was implemented to match the sampling rate of the inertial recordings described later. The resampled x- and y- coordinates were low-pass filtered at 8 Hz using a 4^th^ order zero-lag Butterworth filter.

The 21 known points on the 3D calibration object were manually digitised from both camera views, and the 11 direct linear transformation coefficients required to calculate the multi-view 2D to 3D coordinate transformation^42^ were estimated using an in-house python implementation ported from our open-source Java implementation (https://github.com/tjrantal/Direct-Linear-Transformation). 3D coordinates of the knee and the ankle were then calculated for each sample of the synchronised and low-pass filtered digitised camera view coordinates.

**Fig. 5:**
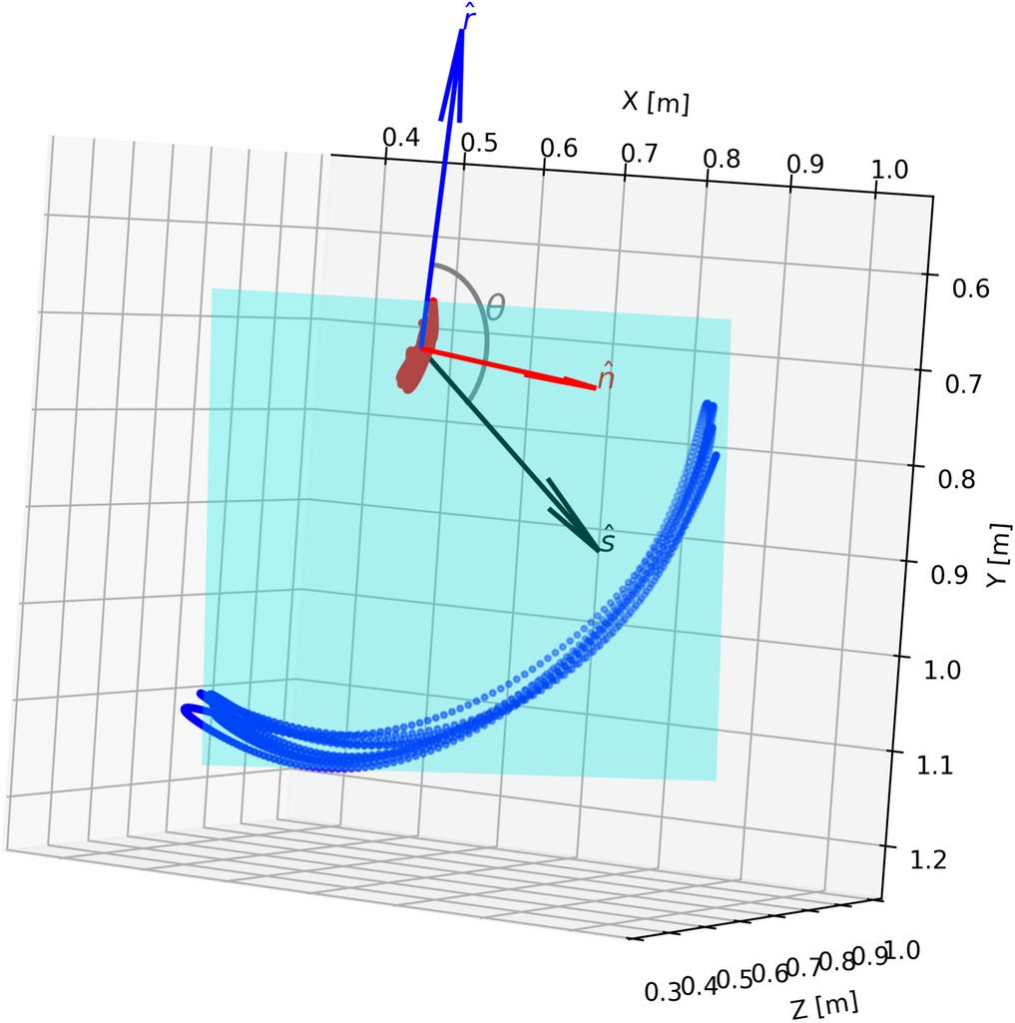
Visualisation of the shank direction angle computation. Sampled knee (red dots) and ankle (blue dots) coordinates are drawn in a 3-dimensional coordinate system. The movement plane determined as a minimum summed squares plane fit onto the knee and the ankle points visualised with a rectangle shaded cyan. Plane normal vector ($\hat{n}$) used as the screw axis indicated with a red arrow. Arbitrary reference vector ($\hat{r}$) within the movement plane indicated with a blue arrow, and shank vector ($\hat{s}$) indicated with black arrow. Shank direction angle ($\theta$) was computed as the angle from $\hat{r}$ to $\hat{s}$ around the screw axis ($\hat{n}$).

Shank angular kinematics were estimated as the angle between shank vector ($\hat{s}$) and an arbitrary reference vector ($\hat{r}$) in the movement plane using the normal of the movement plane ($\hat{n}$) as the screw axis (Fig. 5) as explained next. First, a shank vector ($s$) was determined by subtracting the ankle coordinate from the knee coordinate at each sample instant. The shank vectors were normalised to unit vectors ($\hat{s}$). The movement plane was then defined for the particular exercise set by extracting the knee and ankle point 3D coordinates (e.g., the point cloud comprising the red and the green trace in the top right-hand column of Fig. 4) of the central repetition portion of the synchronised exercise set (experimentally chosen 0.2 to 0.7 relative duration) and fitting a plane onto the point cloud. The central portion was used as the initial and the final parts of the time series may include movements other than the intended exercise.

The movement plane was solved using a minimum sum of squared distances approach to estimate the normal to the plane of movement ($n$) from the plane equation (1):

1 $$\begin{aligned} \textbf{n} \cdot \textbf{X} + D = 0 \end{aligned}$$

where $n$ is the unknown normal of the plane, $X$ are the mean-centred knee and ankle coordinates, and $D = 0$ because a mean offset of the plane from the origin is zero for a mean-centred point cloud. In practice, $n$ was solved using eigenvalue decomposition (NumPy linear algebra ‘eigh‘ function), with the eigenvector corresponding to the minimal eigenvalue used as $n$ and normalised to be a unit vector $\hat{n}$. The cross product $\hat{n} \times (0,0,1)$, normalised to a unit vector, was used as an arbitrary reference vector in the movement plane $\hat{r}$. Shank angle $(\theta )$ was thereafter calculated as the angle between $\hat{r}$ and $\hat{s}$, with $\hat{n}$ as the screw axis (Fig. 5). The NumPy Python library *atan2* function was used to calculate $\theta$ using equation (2):

2 $$\begin{aligned} \theta = \text {atan2}\left( \det \left( [\hat{s}\ \hat{r}\ \hat{n}]\right) ,\ \hat{s} \cdot \hat{r}\right) \end{aligned}$$

Shank angle was thereafter low-pass filtered at 8 Hz using a zero-lag 4^th^ order Butterworth filter. Shank angular velocity was calculated as the differential of shank angle multiplied by the 50 Hz resample rate. Shank angular velocity was also 8 Hz low-pass filtered using a zero-lag 4^th^ order Butterworth filter prior to further analysis. Note that shank angle is not defined in the anatomical sagittal plane, but in the trial-specific major movement plane obtained from the knee and ankle trajectories. This approach helps account for small deviations in limb path or camera alignment in the aquatic environment. Moreover, sign of shank angle and magnitude of the angle are determined by the choice of the coordinate system, and the reference vector, respectively, both of which are arbitrary.

In summary, the shank kinematics estimation processing required the following steps:

1. Extract 2D anatomical key-points (ankle, knee) using MMPose from each camera independently
2. Synchronize video frames between cameras using cross-correlation of ankle trajectories
3. Triangulating synchronized 2D coordinates from both camera views to explicitly reconstruct 3D coordinates using calibrated camera parameters
4. Filtering and smoothing reconstructed 3D coordinates
5. Calculating major movement plane shank kinematics explicitly from filtered 3D ankle and knee positions

### IMU sensor angular kinematics

A single IMU (mounted on participants during exercise sessions using Velcro straps on the right ankle just above the malleoli) sampled 3D acceleration and angular velocity at 50 samples per second with a range of ±16 G and ±2000 deg/s, respectively, using a 16-bit analogue-to-digital conversion. Session data were logged into the sensor internal memory for the duration of the exercise testing protocol and subsequently uploaded onto a portable Android device using a custom data-collection application (Data Collector 3, Kaasa Solutions GmbH). Epochs of interest to this analysis were manually identified by combining the protocol with the recorded signals. Only angular velocity was used in quantification with the accelerations discarded from further consideration.

The orientation of the sensor with respect to the body segment was made irrelevant by leveraging the uniplanar nature of the exercises of interest by the following dimensionality reduction procedure. The recorded angular velocities were centred at zero (i.e., the mean of the signal was removed for each of the axes independently ($\bar{g}$), and the mean centred data were subsequently dimensionality reduced by using the first PCA coefficients^47^ to produce a 1-dimensional angular velocity array. The 1-dimensional angular velocity array was used in subsequent quantification to represent IMU-based shank angular velocity. The described PCA approach captures the primary rotational action executed during the exercise. The IMU-based shank angle was calculated with trapezoidal cumulative integration of the IMU-based angular velocity divided by the 50 Hz IMU sample rate. Integration drift was minimised by high-pass filtering the IMU-based shank angle at 0.05 Hz using a 4^th^ order zero-lag Butterworth filter prior to further analysis. Notably, shank angular velocity and angle are left with an arbitrary sign due to the PCA-based dimensionality reduction. The signs were matched manually between methods for each exercise set based on visual inspection.

### Exercise quantification

Following the processing steps outlined above, a dataset containing segmented movement data from IMU and pose estimation analysis for each participant was obtained. This allowed for subsequent quantification of movement performance. The same exercise quantification procedure was applied to obtain both pose estimation and IMU-based shank angles and angular velocities (described in the previous section). The performance metrics extracted from the data were; repetition range of motion, angular velocity, and duration. Repetition extension and flexion phases were treated separately during the analysis process. Repetition phases were identified using a signal-adaptive threshold approach applied to the shank angle time series. This is equivalent to setting a minimum peak height for flexion and a complementary threshold for extension, ensuring that only true flexion and extension extrema were captured (Supplementary Fig. 1). This process developed by experimentation was as follows; Firstly, the flexion and extension phases of each repetition were identified from the shank angle time series. To achieve this, the signals were mean-centred (mean subtracted), values sorted and the max and the mean of the central 80% of values were identified to isolate a primary data range. Exclusion of the bottom and top deciles made data range estimation more robust. From this subset, thresholds were calculated by determining the median of values near 80% of the max and min, respectively, scaling these medians, and adjusting them relative to the original mean. This resulted in two thresholds that account for data distribution and exclude noise (Supplementary Fig. 1). These values included the maximum amplitude extension and flexion within the set. Flexion repetition phases were then defined as the consecutive negative to positive extrema and extension repetition phases the consecutive positive to negative extrema.

Instances where points of detected negative extrema were not followed immediately by positive extrema (and vice-versa) were not included in subsequent quantification. Range of motion of the repetition phase (flexion or extension) for each repetition was defined as the difference in shank angle between the extrema, repetition angular velocity as the mean angular velocity between the extrema, and phase duration as the number of samples from extrema to extrema divided by sample rate. The median of repetition phases were calculated for an exercise set per participant and used in statistical analyses. Metrics were summarized across repetitions within exercise sets using the median to robustly characterize typical performance; a practical approach in scenarios involving repeated movements (e.g., rehabilitation or training contexts). This method mitigates the influence of anomalous repetitions without arbitrary data exclusion.

### Statistical analysis

Mean and standard deviation are reported to describe mean characteristics and the respective dispersions, where applicable. Criterion validity was evaluated using the intraclass correlation coefficient (ICC) computed for consistency with fixed single raters (ICC(3,1)). Agreement based on ICC was categorised into poor (ICC < 0.50), moderate (0.50 $\ge$ ICC < 0.75), good (0.75 $\ge$ ICC < 0.90), and excellent (ICC $\ge$ 0.90)^48^. The agreement between methods was visualised using Bland-Altman plots, and absolute agreement between the methods was evaluated by computing the mean bias and by comparing the methods using two-sided paired t-tests. The flexion and extension phase results were pooled for the ICC evaluation and Bland-Altman plots. The absolute values rather than signed values were used in the evaluations to enable pooling of both exercise phases, which had different signs. That is, without taking the absolute, the data distributions would have been bimodal for angular velocity and ROM. Taking the absolute resulted in a unimodal distribution appropriate for ICC computations. The statistical tests used in the present examination depend on normal data distribution. Data distributions were visually examined using Q-Q plots and sensitivity analyses were executed where distributions appeared potentially skewed. Potentially skewed distributions were observed for knee flexion-extension phase angular velocity and duration (three participants apparently outlying) as well as hip flexion-extension angular velocity and phase duration due to apparent outliers (one apparent outlier). These violations of the normality assumption were explored in sensitivity analyses by eliminating the apparent outliers, which normalised the distributions. Statistical testing was completed using Pingouin (version 0.5.5) Python library.

As mentioned in the results, these violations of normality did not affect the overall findings. One participant performed the movements significantly slower than the rest of the cohort, which appeared as an outlier with phase durations greater than 2.5 seconds and another performed the leg swings significantly faster than the rest of the cohort. Findings remained the same in a sensitivity analysis without considering their result, confirming our primary results were not driven by outliers, maintaining high agreement between measurement systems. Another apparent outlier was evident in knee extension-flexion exercise where > 30 $^{\circ }$/s difference between methods was observed. Visual inspection of the IMU signals appeared as though the sensor may have been pinched and flicked by the fin worn to provide resistance for the exercise with conspicuous spikes visible in the recorded angular velocity not apparent in any other recording. Sensitivity analysis with this participant removed did not change the findings

## Data Availability

The datasets generated and/or analysed during the current study are not publicly available due to participant privacy considerations under the European General Data Protection Regulation (GDPR). Data are, however, available from the corresponding author, E. P. McShane (eunan.mcshane@goodboost.org), on reasonable request.
